# The challenges and promises of allogeneic mesenchymal stem cells for use as a cell-based therapy

**DOI:** 10.1186/s13287-015-0240-9

**Published:** 2015-12-01

**Authors:** Jun Zhang, Xiaowen Huang, Haijun Wang, Xiaoyan Liu, Tao Zhang, Yunchuan Wang, Dahai Hu

**Affiliations:** Department of Burns and Cutaneous Surgery, Xijing Hospital, Fourth Military Medical University, Xi’an, Shaanxi 710032 People’s Republic of China; Department of Burn and Plastic Surgery, the 205th Hospital of People’s Liberation Army, Jinzhou, Liaoning 121000 PR China; Changchun Zhongyan Medical Cosmetic Hospital, Changchun, Jilin 130000 PR China; Department of Plastic Surgery, General Hospital of Shenyang Military Area Command of People’s Liberation Army, Shenyang, Liaoning 110016 PR China; Department Of Neurology, the 303th Hospital of People’s Liberation Army, Nanning, Guangxi 530021 PR China

## Abstract

Mesenchymal stem cells (MSCs) are ideal for cell-based therapy in various inflammatory diseases because of their immunosuppressive and tissue repair properties. Moreover, their immunosuppressive properties and low immunogenicity contribute to a reduced or weakened immune response elicited by the implantation of allogeneic MSCs compared with other cell types. Therefore, implantation of allogeneic MSCs may be a promising cell-based therapy. In this review, we first summarize the unique advantages of allogeneic MSCs for therapeutic applications. Second, we critically analyze the factors influencing their therapeutic effects, including administration routes, detection time-points, disease models, differentiation of MSCs in vivo, and timing and dosage of MSC administration. Finally, current approaches to allogeneic MSC application are discussed. In conclusion, allogeneic MSCs are a promising option because of their low immunogenicity and immunosuppressive and tissue repair capabilities. Further investigations are needed to enhance the consistency and efficacy of MSCs when used as a cell-based therapy in inflammatory diseases as well as for tissue repair.

## Introduction

Mesenchymal stem cells (MSCs) are classified into various groups according to the cell source, such as bone marrow-derived MSCs (BM-MSCs), adipose-derived MSCs (ASCs), and umbilical cord MSCs. These MSC types share common features, which have been described by the International Society for Cellular Therapy. The minimum criteria for defining MSCs are that they: (a) remain plastic-adherent under standard culture conditions; (b) express CD105, CD73, and CD90 and fail to express CD45, CD34, CD14 or CD11b, CD79a or CD19, and major histocompatibility complex (MHC) class II molecules; and (c) differentiate into osteoblasts, adipocytes, and chondrocytes in vitro [1].

The following unique properties appear to make MSCs ideal for cell-based therapy in various diseases. First, they have multilineage potential, differentiating into various cell types, including adipocytes, hepatocytes, and neurocytes [2–4]. This makes them useful as seed cells to replace damaged tissue in tissue engineering applications. Second, they alleviate tissue injury and promote tissue repair by their anti-apoptotic and cytoprotective effects and angiogenic capacity [5, 6]. Third, they have become a promising approach to treat graft-versus-host disease (GVHD) and autoimmune disease because of their immunomodulatory properties and low immunogenicity [7–9].

## Advantages of allogeneic MSCs for therapeutic applications

Autologous MSC (auto-MSC) applications have some potential limitations. First, it is difficult to obtain sufficient auto-MSCs from some patients—for example, ASCs from thinner patients or BM-MSCs from myelofibrosis patients. Second, MSCs isolated from elderly donors have decreased biological activity, including differentiation and regenerative potential [10, 11], resulting in disappointing treatment outcomes. Third, some systemic diseases, such as diabetes [12], rheumatoid arthritis [13], and systemic lupus erythematosus (SLE) [14], alter the intrinsic properties of MSCs, thus impairing their protective function. It is difficult to obtain sufficient quantities of healthy auto-MSCs with high activity from patients with these diseases. MSC implantation in these patients is therefore challenging. Obtaining allogeneic MSCs (allo-MSCs) from young healthy donors is a reasonable approach to resolving this issue.

Furthermore, auto-MSC extraction is time-consuming, making it difficult to use them promptly to treat acute diseases such as stroke and myocardial infarction. In contrast, allo-MSCs are readily available and can be administered immediately. In addition, commercial allo-MSC production should guarantee quality control and reduce the cost of cell therapies. Therefore, allo-MSCs are promising alternatives to auto-MSCs, with advantages with regard to time, cost, and quality assurance.

Above all, the immunosuppressive properties and low immunogenicity of allo-MSCs contribute to a reduced immune response after implantation. The following mechanisms are responsible for their immunosuppression and low immunogenicity. First, their expression of a low or modest level of MHC class I molecules and lack of expression of MHC class II and co-stimulatory molecules, such as CD40, CD80 (B7-1), and CD86 (B7-2), leads to low immunogenicity, thus avoiding immune responses in recipients [15]. Second, MSCs inhibit the activity of various immune cells, including T cells, B cells, natural killer cells, and dendritic cells via cell–cell contacts and soluble factors [16, 17].

## Factors influencing the protective effect of allo-MSCs

The concept that allo-MSCs may have equivalent efficacy to auto-MSCs has become well established. Increasingly, however, in vivo studies report that allo-MSCs are not fully immune privileged and probably cause an immune response despite the immunosuppressive properties and low immunogenicity of MSCs being documented both in vivo and in vitro. Currently, different research groups have obtained inconsistent or even contradictory results on the therapeutic effects of allo-MSCs in various studies [18–21]. Therefore, the in vivo immunogenicity of allo-MSCs and the relationship between immunogenicity and their protective effects remains to be determined. In addition, the cause of the inconsistent results has yet to be established. We describe in detail the factors that influence the therapeutic effects of allo-MSCs below.

### Administration routes versus therapeutic effects

The routes of MSC administration are classified into two categories: systemic and topical. Some studies have reported that the administration route of allo-MSCs determines the extent of their protective effects.

There are two types of topical administration: intralesional injection (e.g., intracranial, intracerebral, subcutaneous) and local vascular injection (e.g., superior vena cava, mesenteric blood vessels, coronary artery). Compared with systemic administration, topical administration routes may have a common advantage in that MSCs arrive directly at the target tissue with little loss during migration [18, 22]. It was demonstrated that allo-MSCs loaded onto cancellous bone granules have a similar efficacy to auto-MSCs for bone regeneration in bone defect models [23]. Acar et al. [24] reported that direct injection of allo-MSCs into marrow cavities (i.e., intra-bone marrow delivery) had similar effects to intravenous (IV) injection in irradiation-damaged bone marrow repair. However, Gu et al. [25] reported that allo-MSCs implanted via the intrapancreatic route had a greater effect on hyperglycemia correction and increasing insulin secretion in the serum of diabetic rats than those administered via the IV route.

Types of systemic administration include IV, intra-arterial, and intraperitoneal injection. IV is the most common method in preclinical and clinical settings because of its convenience. However, MSCs administered via this route are more easily trapped in small lung capillaries because of their larger size and expression of cell adhesion molecules [26, 27]. Lung entrapment of MSCs decreases the number of MSCs delivered to target tissues and can result in ineffectual treatment [28]. However, some reports have shown that auto-MSCs delivered via IV injection have protective effects in various animal models even when lung entrapment occurs [3, 29]. Similar to auto-MSCs, IV administration of allo-MSCs improved islet function and corrected hyperglycemia without immune rejection in a diabetic rat model [25]. In a rat ischemic stroke model, allogeneic ASCs and BM-MSCs delivered via IV injection decreased cell death, increased cellular proliferation, and improved the functional recovery of the brain [3].

Administration routes determine the microenvironments that MSCs first encounter after entering the patient’s body, thus influencing their differentiation, immunogenicity, and survival [30]. However, the mechanisms responsible for these effects are far from clear because of the limited number of studies performed, and it is necessary to investigate which administration routes of MSCs are best for the diverse range of disease models.

### Evaluation time-points versus therapeutic effects

Short-term (i.e., within a month) but not long-term protection has usually been evaluated in most studies that demonstrate the protective effects of MSCs [29, 31]. In contrast, most studies evaluating their long-term effect have shown no or limited protection [32, 33]. Therefore, the different time-points used in these investigations probably contribute to their different conclusions on the protective effects of MSCs. As MSCs have low immunogenicity but are not fully immune privileged in vivo, immune rejection of allo-MSCs is induced. However, this is too weak to eliminate them immediately, so allo-MSCs can survive for a short period after transplantation. Therefore, they can exert a protective and/or immunosuppressive function in the short-term but are less effective in the long-term. More studies into implanted MSCs are urgently needed to simultaneously evaluate their short- and long-term protective effects.

### Disease models versus therapeutic effects

It is well established that allo-MSCs can alleviate GVHD in the setting of allogeneic hematopoietic stem cell transplantation in preclinical [34] and clinical studies [35]. Moreover, the Prochymal brand of remestemcel-L, the first stem cell drug, has been approved for the market. Prochymal is a MSC product prepared from bone marrow aspirates of healthy human donors, and shows potential for treating acute GVHD [19, 36]. In addition to GVHD models, the efficacy and safety of allo-MSCs have been widely documented in autoimmune disease models. Allo-MSCs can reduce the clinical relapse rate and improve the function of damaged organs in models of autoimmune diseases, including SLE and Crohn's disease [9, 37]. The technology available for allo-MSC applications for GVHD and Crohn's disease is currently comparatively mature; of the 13 available clinical trials on Prochymal registered in clinical trials databases, five have been for use in GVHD and Crohn's disease.

Although MSCs display a protective function in GVHD and autoimmune disease models, controversy exists about allo-MSC immunosuppression in the setting of solid organ transplantation [20, 38, 39]. For example, allo-MSCs show no graft protection in many studies [20]. Unexpectedly, some studies have reported that allo-MSCs are ineffective at prolonging allograft survival and tend to cause more rapid—and a greater degree of—immune rejection [20, 21]. Therefore, the use of various disease models may be one reason for the controversy about the protective effects of allo-MSCs.

### Differentiation of MSCs in vivo versus therapeutic effects

The low immunogenicity of MSCs does not ensure they are fully immune privileged in an in vivo setting. Allo-MSC immunogenicity after differentiation can weaken or even inhibit their therapeutic effects. Huang et al. [33] reported that expression of immunogenic MHC-Ia and MHC-II is strongly increased in differentiated MSCs compared with undifferentiated MSCs in a rat myocardial infarction model. The implanted allo-MSCs induced expression of a specific anti-donor alloantibody in serum after differentiation (5 weeks), which limited the long-term (more than 5 months) protective effects of MSCs on the heart. However, allo-MSCs were as effective as auto-MSCs in improving cardiac function for at least 3 months. In a diabetic rat model, Gu et al. [25] reported that implanted allo-MSCs did not express MHC-II and did not trigger cellular cytotoxicity and immune rejection until they differentiated into insulin-producing cells. Even so, the therapeutic effects of allo-MSCs for damaged pancreas were maintained after their differentiation.

From these results, we find that the presence of immunogenicity after differentiation decreases the therapeutic effects of allo-MSCs, although it does not indicate the definite loss of protective effects immediately, which is consistent with previous reports [40, 41]. We speculate on the probable reasons for this. First, even in specific induction conditions in vitro, only some MSCs differentiate; therefore, sufficient allo-MSCs remain in an undifferentiated state to ensure their survival and execute their protective effects on the immune systems of recipients. Second, the immunoreaction is too weak to quickly eliminate differentiated MSCs; a recipient’s immune systems needs some time to eliminate all of the allo-MSCs. Current data on this issue are lacking and the specific protective mechanism that functions after differentiation needs to be further investigated.

### Timing of MSC administration versus therapeutic effect

The immune status of a recipient before and after allograft organ transplantation determines the survival of implanted allo-MSCs. Crop et al. [42] reported that, before kidney transplantation, recipient peripheral blood mononuclear cells (PBMCs) did not lyse allo-MSCs, but that PBMCs isolated 3, 6, and 12 months after transplantation showed increasing ability to lyse allo-MSCs. In vivo experiments have shown that the different timing of auto-MSC transplantation determines their therapeutic effect in a myocardial infarction model [43]. As reported for auto-MSCs, a recent study by Rigol et al. [41] showed that allo-ASCs induce better neovascularization and a better long-term prognosis at 15 min after reperfusion than a week later. In addition, Cho et al. [44] reported that a single injection of MSCs, either systemically or subcutaneously, did not induce a detectable adaptive immune response. However, repeated injection of MSCs into the same site resulted in alloantibody production. Therefore, differences in administration timing have probably led to inconsistent conclusions regarding the immunogenicity and therapeutic effects of allo-MSCs.

### Dosage of MSC administration versus therapeutic effects

Different doses of MSCs have different immune response or protective effects. Allo-MSCs injected intracranially induced transient dose-dependent immune rejection, which reduced MSC engraftment levels and their protective effects [45, 46]. In contrast, an animal study on myocardial infarction by Wolf et al. [47] indicated that allo-MSCs limited myocardial infarct size and improved the functional outcome in a dose-dependent manner. Currently, the relationship between MSC dose and therapeutic effects is far from clear. Therefore, the optimal dose of implanted allo-MSCs needs to be further investigated to maximize their therapeutic function in various disease models.

## Application strategies for allogeneic MSCs

Many unique features make MSCs a promising therapeutic option in tissue repair and immunosuppression. Although the direct application of allo-MSCs has a certain protective effect, various measures taken during or before transplantation can have a great effect on improving treatment outcomes (Table 1).

**Table 1:** Strategies to enhance efficiency of MSC-based treatment

Study	Method	Experimental model	In vitro or in vivo	Cell types	Conclusion
Maccario et al. [64]	Combination cyclosporine and MSCs	–	In vitro	Human BM-MSCs	Enhancement of the immunosuppressive effect of MSCs
Buron et al. [65]	Combination mycophenolate acid and MSCs	–	In vitro	Human BM-MSCs	Enhancement of the immunosuppressive effect of MSCs
Ge et al. [48]	Combination rapamycin and MSCs	Allogeneic cardiac transplantation (mouse)	In vivo	Mouse BM-MSCs	Attenuation of alloimmune responses and promotion of cardiac allograft tolerance
Peng et al. [49]	Combination tacrolimus and MSCs	Allogeneic renal transplantation (human)	In vivo	Human BM-MSCs	Induction of donor-specific graft tolerance and maintaining long-term graft survival and function
de la Garza-Rodea et al. [53]	MSCs transfected with *US11* gene from HCMV	NOD/SCID (mouse)	In vivo	Human BM-MSCs	Down-regulation of MHC class I surface expression and preventing rejection of xenogeneic MSCs
Soland et al. [66]	MSCs transfected with *US6* gene and *US11* gene from HCMV	In utero transplantation of fetuses (sheep)	In vivo	Human fetal liver-derived MSCs	Decreasing recognition of MSCs by the immune system and enhancing engraftment of MSC-US11 and MSC-US6 in fetal sheep liver
Levy et al. [67]	MSCs transfected with *IL-10* gene	Inflammation of ears (mouse)	In vivo	Mouse BM-MSCs	Improvement of immunosuppressive properties and anti-inflammatory effect
Sullivan et al. [54]	MSCs transfected with *Ctla4ig* gene	Inflammatory arthritis (mouse)	In vivo	Mouse BM-MSCs	Improvement of the homing and delaying the onset of inflammatory arthritis
Chen et al. [55]	MSCs transduced with *Cxcr*-*4* gene	Allogeneic bone marrow transplantation (mouse)	In vivo	Mouse BM-MSCs	Promoting recovery of HSCs and hematopoietic organs
Eliopoulos et al. [56]	*Epo* gene enhanced MSCs	Acute renal injury (mouse)	In vivo	Mouse BM-MSCs	Augmenting the protective properties of MSCs and increasing the survival rate of mouse
Yuan et al. [59]	Combination MSCs and hydrogels	Normal rabbit	In vivo	Rabbit BM-MSCs	Augmenting the isolation from the host immune system and attenuating severe immune rejection
Dhingra et al. [58]	Combination MSCs and biodegradable hydrogels that slowly released PGE2	Myocardial infarction (rat)	In vivo	Rat BM-MSCs	Preventing rejection of implanted MSCs and restoring cardiac function
Sarkar et al. [57]	MSCs engineered with PLGA particles containing dexamethasone	–	In vitro	Human BM-MSCs	Controlling the differentiation of particle-carrying cells
Ko et al. [68]	MSCs coated with PPG followed by antibodies to ICAM-1	–	In vitro	Mouse BM-MSCs	Promoting the attachment of MSCs to endothelial cells
Sarkar et al. [69]	MSCs engineered with lipid vesicles to present biomolecular ligands	–	In vitro	Human BM-MSCs	Immobilizing adhesion ligands and promoting the homing of MSCs

*BM-MSC* bone marrow-derived mesenchymal stem cell, *HCMV* human cytomegalovirus, *HSC* hematopoietic stem cell, *ICAM-1* intercellular cell adhesion molecule-1, *MHC* major histocompatibility complex, *MSC* mesenchymal stem cell, *PGE2* prostaglandin E2, *PLGA* polylactide-co-glycolic acid, *PPG* palmitated protein G

### Combined application with immunosuppressants

The co-application of MSCs with immunosuppressants increases their protective effects compared with their separate application. On one hand, immunosuppressants improve the effects of MSCs by prolonging their survival time in allograft organ transplantation and, on the other, MSCs can decrease the side effects of immunosuppressants. For example, Ge et al. [48] observed that the immunosuppressant Rapa enabled successful MSC engraftment by suppressing the immune response to allo-MSCs after heterotopic cardiac transplantation. Moreover, MSCs markedly enhanced the immunosuppressive effect of Rapa, thus enabling the dosage (and side effects) to be reduced [48]. MSCs attenuated acute immune rejection in renal transplantation, and had the potential benefit of reducing the dosage of the conventional immunosuppressant, tacrolimus [49].

### Genetic modification of MSCs

The effectiveness of genetically modified auto-MSCs has been reported in different disease models [50–52]. Similarly, the protective effect of allo-MSCs was improved by gene modification. de la Garza-Rodea et al. [53] observed that BM-MSCs with a modified *US11* gene led to decreasing expression of MHC-1. The *US11* gene modification contributed to evasion of recognition by cytotoxic lymphocytes and extended the persistence of MSCs in the allogeneic host. In contrast to wild-type allo-MSCs, allo-MSCs expressing cytotoxic T lymphocyte associated antigen-4 (CTLA4Ig) demonstrated enhanced inhibition of T-cell responses [54]. The genetically modified MSCs delayed the onset of inflammatory arthritis and decreased the amount of damage in collagen-induced arthritis. Chen et al. [55] reported that MSCs expressing allogeneic C-X-C chemokine receptor type 4 (CXCR-4) promoted a greater level of hematopoietic recovery and sustained hematopoiesis compared with unmodified MSCs. The protection of MSCs resulted from the enhanced ability to home to bone marrow and spleen. Allo-MSCs with modified *Epo* gene significantly increased protective effects for kidney and improved the survival of mice in an acute kidney injury model [56].

### Method of cell engineering

The fate of implanted allo-MSCs is tightly influenced by the microenvironment encountered. Intracellular depots have been generated through cell engineering to provide controlled microenvironments for MSCs. These depots continuously release drugs and cellular factors which affect the homing, viability, differentiation of MSCs, etc. For example, MSCs engineered with poly lactide-co-glycolic acid particles containing dexamethasone promoted the osteogenic differentiation of MSCs [57].

Hydrogels were previously reported to be promising allo-MSC carriers for tissue engineering. Dhingra et al. [58] reported that the use of a biodegradable, temperature-sensitive hydrogel for the slow release of prostaglandin E2 at the cell implantation site could prevent rejection of implanted allo-MSCs and restore cardiac function in a myocardial infarction model. Interestingly, hydrogels themselves have been documented to modulate the immunological properties of allo-MSC tissue-engineered cartilage. Neonatal rabbit allo-MSCs induced lower allogeneic lymphocyte proliferation and reduced the expression of MHC class I and II molecules when seeded in a collagen hydrogel compared with sponge and membrane [59].

Recently, there have been fewer studies on applications of allo-MSCs compared with auto-MSCs. Auto-MSC studies have provided insight into allo-MSC applications. For example, the pre-stimulation of auto-MSCs with interferon-gamma increased their immunosuppressive capacity, reduced mucosal damage, and enhanced their therapeutic efficacy in animal models of colitis [60]. In addition, hypoxia preconditioning is reported to increase the protective effect of auto-MSCs in disease models such as hemorrhagic stroke [61], ischemia [62], and pulmonary fibrosis [63].

## Conclusion and future perspectives

MSCs have shown promise in cell replacement or transplantation for their immunosuppressive and tissue repair effects. However, it is difficult to isolate sufficient quantities of healthy auto-MSCs with high activity from older or thinner people and patients with diabetes, rheumatoid arthritis or SLE. Moreover, auto-MSCs are not suited to the prompt treatment of acute diseases because extraction of them is time-consuming. Because of their immune suppression properties and low immunogenicity compared with other cell types, the implantation of allo-MSCs may, therefore, be more reasonable and appropriate. Although various studies have provided inconsistent conclusions on the therapeutic effects of allo-MSCs, allo-MSCs are still a promising option in immunosuppressive and tissue repair therapy.

To date, we have been unable to obtain consistent results from the insufficient pre-clinical and clinical data on the immunogenicity and protective effects of allo-MSCs. The following issues need to be addressed in further research. First, which immune molecules and cells are involved in the potential immune response? Second, what is the dynamic fate of implanted allogeneic ASCs, including being eliminated by recipients, being maintained in the stem cell state, or differentiating into various cell types? It will be helpful to assess the in vivo efficiency of allo-MSCs compared with that of auto-MSCs. Third, the factors that influence their therapeutic effects and how they result in the present inconsistent results are far from clear. Last, strategies to enhance the consistency and efficacy of allo-MSCs as a cell-based therapy should be investigated in inflammatory diseases as well as for tissue repair.

## Abbreviations

allo-MSC: allogeneic mesenchymal stem cell
ASC: adipose-derived mesenchymal stem cell
auto-MSC: autologous mesenchymal stem cell
BM-MSC: bone marrow-derived mesenchymal stem cell
GVHD: graft-versus-host disease
IV: intravenous
MHC: major histocompatibility complex
MSC: mesenchymal stem cell
PBMC: peripheral blood mononuclear cell
SLE: systemic lupus erythematosus
